# The Efficacy and Safety of Inclisiran for Low-Density Lipoprotein (LDL) in Patients With Atherosclerotic Cardiovascular Disease (ASCVD): A Systematic Review of Randomized Controlled Trials

**DOI:** 10.7759/cureus.70411

**Published:** 2024-09-28

**Authors:** Hyder Mirghani, Bandar H Albalawi, Mohammed S Alshehri, Waseem M Almalawi, Sumaya Alanezi, Mahmoud Alarki, Abdullah A Alyahya, Sultan Alshaman, Mariam S Shaman, Abeer AlAnazi

**Affiliations:** 1 Internal Medicine, University of Tabuk, Tabuk, SAU; 2 Surgery, King Khalid Hospital, Tabuk, SAU; 3 Medicine, University of Tabuk, Tabuk, SAU; 4 Internal Medicine, King Fahad Hospital (KFH), Tabuk, SAU; 5 Medicine and Surgery, University of Tabuk, Tabuk, SAU; 6 College of Medicine, University of Tabuk, Tabuk, SAU; 7 Medicine, College of Medicine, Tabuk University, Tabuk, SAU

**Keywords:** ascvd, atherosclerotic cardiovascular disease, cholesterol, inclisiran, lipid metabolism

## Abstract

This systematic review synthesizes current evidence to evaluate the safety and efficacy of inclisiran for lowering low-density lipoprotein cholesterol (LDL-C). A thorough search was conducted across four main databases - PubMed, Web of Science, SCOPUS, and Science Direct - for relevant studies published in the literature within the last five years (2020-2024). We included seven studies with a total of 6831 participants, of which 4661 (68.2%) were males. The follow-up duration ranged from 60 to 1350 days. The intervention we focused on was a subcutaneous injection of inclisiran (284 mg) in the seven trials.

Of the total population, 2411 (35%) had diabetes mellitus (DM) and 5335 (78.1%) had hypertension (HTN). The seven trials documented a significant reduction in the mean LDL-C level following the inclisiran application. Early inclisiran administration recorded better results. Inclisiran was found to be efficient, safe, and tolerable among atherosclerotic cardiovascular disease (ASCVD) patients. An inclisiran regimen of 284 mg proved to be efficacious and safe for reducing ASCVD. Most of the affected participants were males and hypertensive. Our findings highlight the need for additional clinical trials to examine inclisiran's safety and effectiveness with regard to cardiovascular outcomes. Extended surveillance studies may yield deeper insights into the efficacy, durability, and tolerability of inclisiran.

## Introduction and background

Atherosclerotic cardiovascular disease (ASCVD) is a major contributor to death and disability worldwide. One of the defining features of the conditions is plaque accumulation in blood vessels, leading to decreased blood flow and potentially life-threatening events such as heart attacks and strokes. The management of ASCVD typically involves lifestyle modifications, medications, and in some cases, invasive procedures such as stents or bypass surgery [1]. The use of new drugs for ASCVD risk mitigation has garnered significant attention in recent years. One such medication is inclisiran, which belongs to a new class of drugs known as PCSK9 inhibitors. PCSK9 inhibitors work by targeting a protein in the liver that controls how quickly low-density lipoprotein cholesterol (LDL-C) leaves the bloodstream. Incisiran lowers the amount of LDL-C and lowers the likelihood of ASCVD events by blocking this protein [2].

Numerous, extensive clinical trials have been carried out to assess the safety and effectiveness of inclisiran in lowering the risk of ASCVD. In the ORION trials, which included over 3,000 individuals with a control group on placebo, inclisiran effectively decreased LDL-C levels by up to 50% in individuals with ASCVD or those at high risk for the condition. This decrease in LDL-C persisted throughout the studies, proving inclisiran's long-term effectiveness [3]. Apart from reducing LDL-C levels, inclisiran has also been shown to help lower the chance of ASCVD incidents including strokes and heart attacks. When weighed against a placebo, inclisiran significantly lowered the risk of serious cardiovascular diseases, according to the ORION studies. This demonstrates the potential of inclisiran to improve outcomes in patients with ASCVD [4,5].

While inclisiran has shown promising efficacy for ASCVD risk reduction, it is crucial to consider this medication's possible adverse effects. Flu-like symptoms, increased kidney enzymes, and injection site responses are the common adverse effects of inclisiran. Inclisiran may also, in rare instances, result in allergic reactions or liver issues. Healthcare professionals must keep a close eye out for these possible adverse effects in patients on inclisiran and modify treatment as necessary [6]. Despite advancements in medical therapy, ASCVD remains a major cause of disease and mortality globally. Hence, there is a pressing need to implement innovative treatment strategies that can effectively reduce ASCVD risk with minimal adverse effects. This study aims to summarize the existing evidence on the efficacy and side effects of inclisiran for LDL among ASCVD patients.

## Review

Methods

We conducted this review by adhering to the Preferred Reporting Items for Systematic Reviews and Meta-Analyses (PRISMA) guidelines [7]. To find English-language research on the efficacy of inclisiran, we performed a thorough electronic search of the databases PubMed, Web of Science, SCOPUS, and Science Direct, which revealed a decreased risk of ASCVD. The following relevant keywords were incorporated into the search strategy: “Inclisiran.” “Leqvio,” “PCSK9 inhibitors,” “Atherosclerosis,” “Atherosclerotic cardiovascular disease,” “Safety,” “Complications,” and “Efficacy.” A pair of impartial evaluators methodically examined the search outcomes, identified relevant research, collected pertinent information, and evaluated the caliber of the included studies by utilizing suitable assessment instruments.

Eligibility Criteria

The inclusion and exclusion criteria are summarized in Table 1.

**Table 1:** Eligibility criteria for the inclusion of the studies ASCVD: atherosclerotic cardiovascular disease

Inclusion criteria	Exclusion criteria
Studies must be published in English	Non-English language studies
Research must involve human participants and be categorized as randomized controlled trials	Animal studies and in vitro studies
Studies must evaluate the efficacy and/or side effects of Inclisiran for ASCVD risk reduction	Case reports, conference abstracts, editorials, letters, and commentaries that do not focus on inclisiran
Research must provide a clear description of inclisiran intervention and relevant outcomes related to cardiovascular disease risk reduction	Studies that lack clear descriptions of inclisiran intervention or related cardiovascular outcomes
Studies must present available data on efficacy outcomes such as cardiovascular events, mortality, lipid levels, or other relevant markers	Research with insufficient data on efficacy or safety outcomes
Studies must include data on safety outcomes such as reported side effects, adverse events, or discontinuations related to inclisiran therapy	Studies exhibiting inadequate methodological quality or significant risk of bias

Data Extraction

To guarantee accuracy, Rayyan (QCRI) was used to validate the search results [8]. The research team carefully examined all of the papers that met the inclusion criteria. Any disagreements were settled by way of consensus. Important research details, such as study names, authors, publication year, location, subject demographics, gender distribution, population type, follow-up duration, intervention, prevalence of diabetes mellitus (DM), prevalence of HTN, baseline LDL-C, LDL-C after treatment, and its primary results, were documented by using a pre-designed data extraction form. To evaluate the possibility of bias, an impartial assessment instrument was used.

Data Synthesis

To provide a qualitative evaluation, summaries of the research findings and components were prepared using data from relevant studies. The most effective way to use the data from the research included in the review was determined once the systematic review's information gathering was complete.

Risk of Bias Assessment

The risk of bias in the controlled studies that were included was evaluated using the Cochrane Collaboration Risk of Bias (ROB) tool (2011). The outcomes are presented in a table using several color schemes in the Results section below. A substantial risk of bias is indicated by red, a low risk by green, and an inability to identify the risk owing to inadequate information by yellow.

Results

Search Results

**Figure 1:**
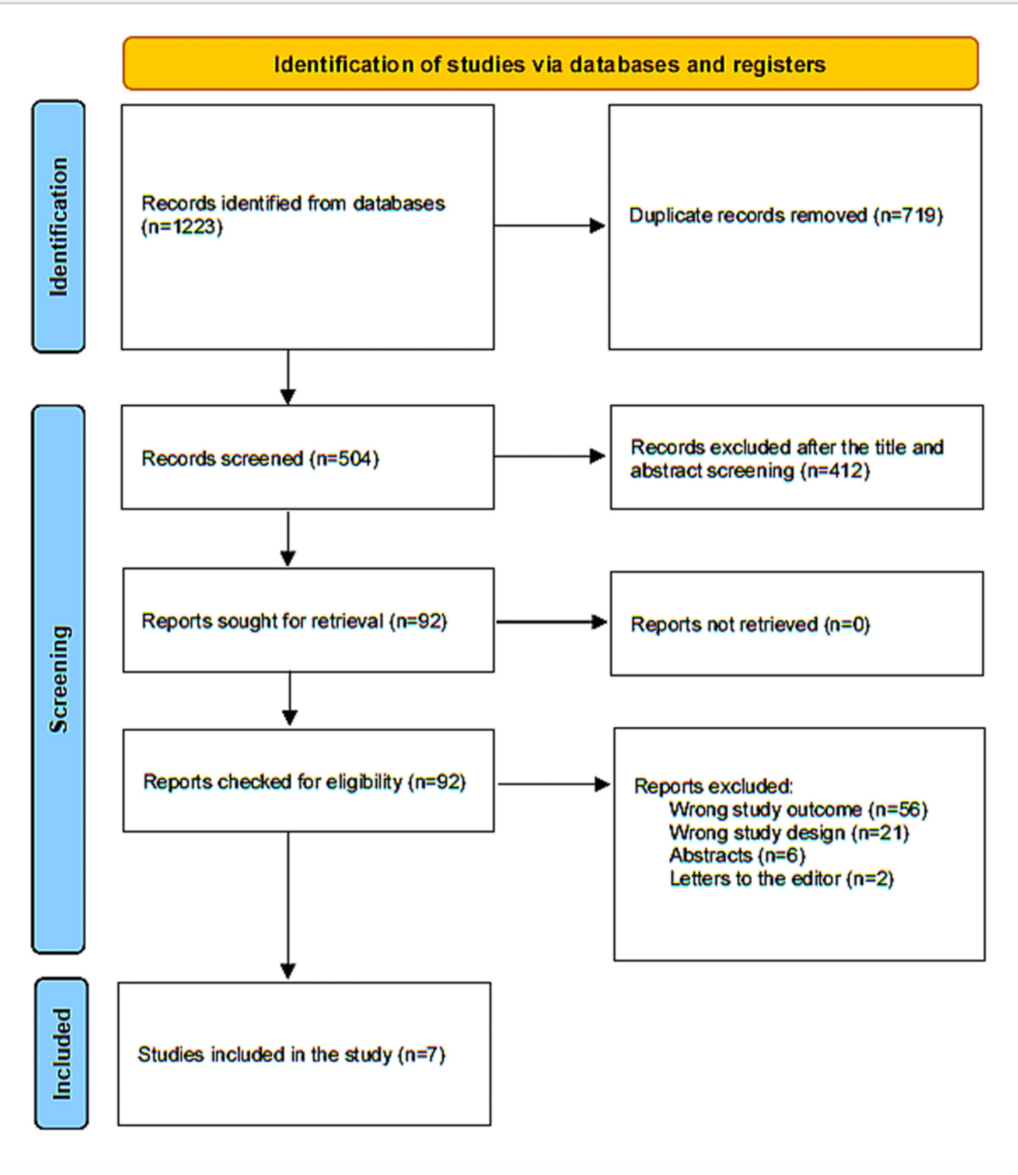
PRISMA flowchart depicting the selection of studies PRISMA: Preferred Reporting Items for Systematic Reviews and Meta-Analyses

A comprehensive search initially elicited 1223 research papers, of which 719 were duplicates and were removed. Of the remaining 504, 412 papers were excluded after their titles and abstracts were examined. Every report that was sought was located. After 92 publications were screened for full-text evaluation, 56 were discarded because the study results were inaccurate, 21 because the study designs were inappropriate, six as they were abstracts, and two articles were letters to the editor. Ultimately, seven research papers that fully met our were included in this systematic review. A synopsis of the study selection process is presented in Figure 1. 

Sociodemographics

Table 2 shows the sociodemographic details of the cohorts in the included studies. Of the 6831 individuals in the seven trials, 4661 (68.2%) were men. Every study that was included was a randomized controlled experiment [9-16]. Four studies were conducted in the US [10,12,13,15], one was a multicentric one from Asia [11], one was performed in the UK [14], and one in South Africa [16]. The earliest study was carried out in 2020 [15] while the most recent one was conducted in 2024 [10,11]. 

**Table 2:** Sociodemographic characteristics of cohorts in the included studies

Study	Study design	Country/region	Participants	Mean age, years	Males, n (%)
Koren et al., 2024 [10]	Randomized controlled trial	USA	Inclisiran first (n=225) and usual care (n=225)	67	311 (69.1%)
Huo et al., 2024 [11]	Randomized controlled trial	Asia	Inclisiran (n=170) and placebo (n=174)	59.5 ± 10.9	257 (74.7%)
Wright et al., 2021 [12]	Randomized controlled trial	USA	Inclisiran (n=1,833) and placebo (n=1,827)	64.1 ± 9.98	2470 (67.5%)
Ray et al., 2023 [13]	Randomized controlled trial	USA	380	63	243 (64%)
Padam et al., 2022 [14]	Randomized controlled trial	UK	80	64 ± 10.3	52 (65%)
Ray et al., 2020 [15]	Randomized controlled trial	USA	Inclisiran (n=810) and placebo(n=807)	64.8 ± 8.3	1160 (71.7%)
Raal et al., 2022 [16]	Randomized controlled trial	South Africa	Inclisiran (n=148) and placebo (n=150)	58.3 ± 10.3	168 (56.4%)

Clinical Traits and Outcomes

Table 3 demonstrates the clinical traits. There were ASCVD patients in six of the seven trials [10,11,13-16] and only one included patients with familial hypercholesterolemia or atherosclerosis [12]. The length of the follow-up ranged from 60 days [14] to 1350 days [13]. The intervention involved a subcutaneous injection of inclisiran (284 mg) in the seven trials. Of the total population, 2411 (35%) had DM and 5335 (78.1%) had HTN. The seven trials recorded a noteworthy decline in the average level of LDL-C after following the inclisiran application. Early inclisiran administration recorded better results [10]. Inclisiran was found to be efficient, safe, and tolerable in the included patients [11-16].

**Table 3:** Clinical characteristics and outcomes of the participants in the included studies ASCVD: atherosclerotic cardiovascular disease; DM: diabetes mellitus; HTN: hypertension; LDL-C: low-density lipoprotein cholesterol; PCSK9: proprotein convertase subtilisin/kexin type 9; siRNA: small interfering RNA

Study	Population type	Follow-up, days	Intervention	DM, n (%)	HTN, n (%)	Baseline LDL-C, mg/dL	LDL-C after inclisiran, mg/dL	Main outcomes
Koren et al., 2024 [10]	ASCVD patients	330	Inclisiran 284 mg	190 (42.2%)	410 (91.1%)	97.4	<70	These findings emphasize how crucial it is to begin initiating inclisiran earlier in the course of treatment and the pressing need to enhance standard care for ASCVD patients
Huo et al., 2024 [11]	ASCVD patients	330	Inclisiran 284 mg	145 (42.2%)	223 (64.8%)	109	55.4	Over time, inclisiran may aid in reducing long-term exposure to increased LDL-C. Incisiran was effective and well tolerated when used in conjunction with diet and the highest possible statin dosage for Asian people with ASCVD or high risk of ASCVD and increased LDL-C, including those with heterozygous familial hypercholesterolemia
Wright et al., 2021 [12]	Patients with familial hypercholesterolemia or atherosclerosis	540	Inclisiran 284 mg	1318 (36%)	2919 (79.8%)	111.9	61.2	When used twice a year, inclisiran lowers LDL-C by an average of 50.7% and is well tolerated, except for moderate injection site reactions and bronchitis, which are mostly self-limiting
Ray et al., 2023 [13]	ASCVD patients	1350	Inclisiran 284 mg	87 (23%)	248 (67%)	59.9	28.3	Over a 4-year period, inclisiran administered subcutaneously twice a year produced reductions in PCSK9 and LDL cholesterol levels that are substantial and prolonged. This dosage was also well tolerated. siRNA-based treatments are secure and may offer a practical means of long-term risk factor management, such as LDL-C
Padam et al., 2022 [14]	ASCVD patients	60	Inclisiran 284 mg	19 (23.8%)	32 (40%)	63	32.4	When using inclisiran per the criteria set forth, LDL-C was considerably lower after two months, according to the National Institute for Health and Care Excellence, and the efficacy was comparable to that shown in trials with good tolerability
Ray et al., 2020 [15]	ASCVD patients	510	Inclisiran 284 mg	568 (53.1%)	1301 (80.5%)	107.2	53.5	It was possible to achieve a considerable 50% reduction in LDL-C levels with an inclisiran regimen every six months. Inclisiran caused more injection-site adverse effects than a placebo
Raal et al., 2022 [16]	ASCVD patients	510	Inclisiran 284 mg	84 (28.2%)	202 (76.8%)	64.8	32.4	LDL-C was effectively reduced and the inclisiran medication was well tolerated. These numbers line up with the information provided for the global ORION cohort

Risk of Bias Assessment

**Figure 2:**
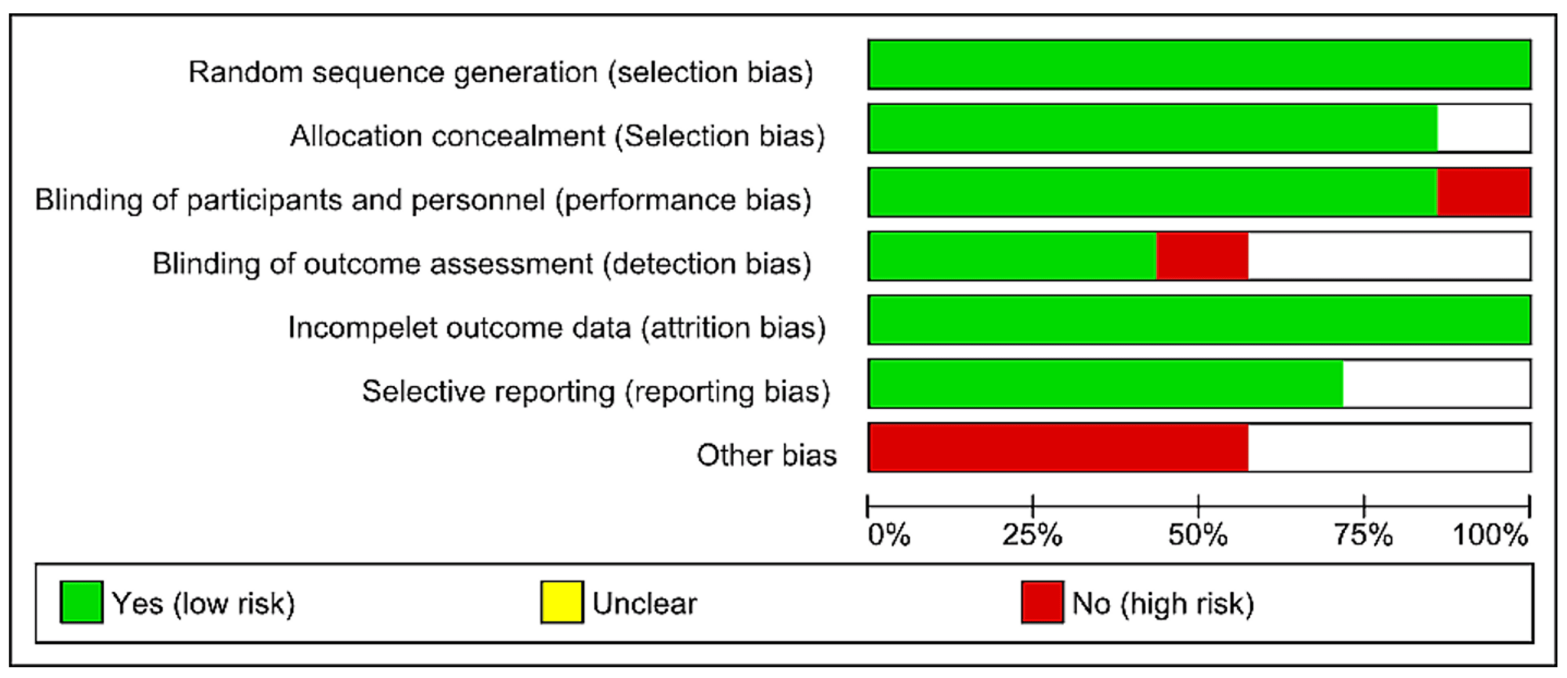
Risk of bias graph

**Figure 3:**
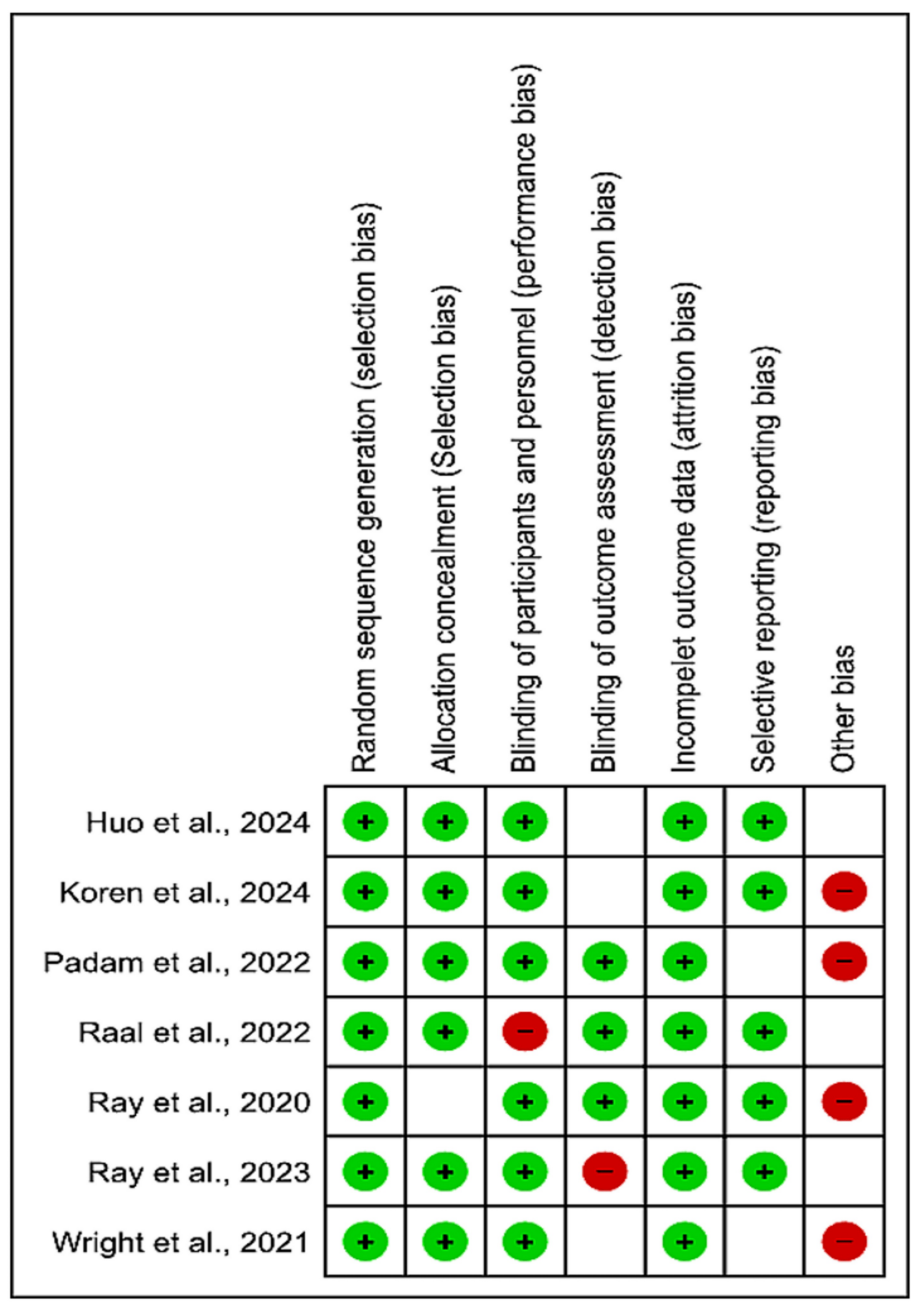
Risk of bias summary Huo et al., 2024 [11]; Koren et al., 2024 [10]; Padam et al., 2022 [14]; Raal et al., 2022 [16]; Ray et al., 2020 [15]; Ray et al., 2023 [13]; Wright et al., 2021 [12]

Figures 2-3 present the risk of bias assessment for the included studies; most of the studies had a low risk of bias.

Discussion

In patients with an elevated risk of cardiovascular atherosclerosis, inclisiran is a potentially effective medication for decreasing LDL-C levels. Its reasonable safety profile and twice-yearly dosage regimen make it an attractive option; it is a well-tolerated and practical alternative for individuals who find it difficult to adhere to regular pharmaceutical regimens. While it has been demonstrated to help decrease LDL-C, further research is still needed to determine how it influences the decrease in cardiovascular events [17]. 

We assessed the impact of inclisiran in lowering the incidence of ASCVD and related mortality in this systematic analysis, which included seven RCTs with 6831 patients in total. Our findings indicate that the use of inclisiran was linked to a noteworthy decrease in the mean LDL-C level. Early inclisiran administration recorded better results [10]. Inclisiran was found to be efficient, safe, and tolerable [11-16]. A comparable meta-analysis and systematic review by Dutta et al. revealed that inclisiran had remarkable effectiveness in managing dyslipidemia, along with a reduced likelihood of noteworthy adverse cardiovascular events [18]. Cowart et al. have reported that for patients who have difficulty administering PCSK-9 inhibitors or self-injecting, inclisiran is a viable substitute. A healthcare provider will deliver inclisiran maintenance therapy twice a year [19]. As per Talasaz et al., inclisiran outcome trials suggest a class effect among PCSK9-targeting medications in terms of lowered risk of myocardial infarction (MI) and LDL-C. Data from trials using inclisiran and alirocumab support a decrease in a composite of MI, cardiovascular mortality, and stroke in patients with confirmed ASCVD. While some individuals may experience injection site reactions, PCSK9-targeting therapies seem to be well tolerated with little chance of major side effects [20].

Of the total population in our included studies, 2411 (35%) had DM and 5335 (78.1%) had HTN. After internalization, PCSK9 attaches increases the quantity of LDL-C in the circulation and decreases the amount of LDL-C absorbed by the liver by binding to the LDL receptors on the surface of hepatocytes and degrading them. PCSK9 medications reduce plasma PCSK9, which in turn reduces major cardiovascular events and has lipid-lowering effects [21]. These substances successfully lowered the levels of non-HDL-C, lipoprotein (a) [Lp(a)], and amino acid apolipoprotein B (apoB). Both inclisiran and PCSK9 monoclonal antibodies showed the ability to reduce lipids in addition to LDL-C [22]. Additionally, people with type 2 DM (T2DM) are more likely to meet the prescribed LDL-C targets than those without the disease, both with and without atherogenic dyslipidemia [23,24].

Statins are currently the first-line treatment for ASCVD prevention and treatment, along with non-pharmaceutical lifestyle changes. Hence, current guidelines suggest that patients with hypercholesterolemia should start taking a statin; nevertheless, many patients require extra LDL-C-lowering drugs, especially those with high or extremely high cardiovascular risk [25,26]. However, long-term adherence to statin medication is not recommended [27]. A synthetic, long-acting siRNA called inclisiran is used in RNAi-based therapeutics; in general, these therapies do not require as frequent a dosage as RNA-based treatments such as antisense oligonucleotides or antibody therapy [28].

This systematic review has several limitations that must be acknowledged. Firstly, the fact that only randomized controlled trials were included may limit the generalizability of findings. Additionally, the potential for publication bias exists, given that studies with positive outcomes are more likely to be published. Variations in trial design, dosage, and follow-up durations among the included studies could introduce heterogeneity, complicating the comparison of results. Lastly, while adverse event data were captured, the reporting quality varied across trials, which may lead to underestimations or overestimations of inclisiran's safety profile.

## Conclusions

A regimen of inclisiran 284 mg proved to be efficacious and safe for reducing ASCVD. Most of the affected participants were males and hypertensive. Our findings emphasize the need for additional clinical trials to examine inclisiran's safety and effectiveness with regard to cardiovascular outcomes. Extended surveillance studies may yield further, comprehensive information on inclisiran's effectiveness, resilience, and tolerability.
